# 低危骨髓增生异常综合征患者应用低氧诱导因子脯氨酰羟化酶抑制剂治疗贫血的疗效和安全性分析

**DOI:** 10.3760/cma.j.cn121090-20230825-00089

**Published:** 2024-03

**Authors:** 悦 吕, 赠华 林, 力 杨, 红 刘

**Affiliations:** 南通大学附属医院血液内科、南通大学医学院，南通 226001 Department of Hematology, Affiliated Hospital of Nantong University, Medical School of Nantong University, Nantong 226001, China

## Abstract

骨髓增生异常综合征是起源于造血干细胞的一组异质性髓系肿瘤性疾病，表现为骨髓病态造血和高风险向急性髓系白血病转化。低危患者的治疗主要是改善造血，提高生活质量。罗沙司他是全球首个口服小分子低氧诱导因子脯氨酰羟化酶抑制剂，与传统红细胞生成素不同，罗沙司他通过多种途径纠正贫血。本研究回顾性分析罗沙司他使用前后低危骨髓增生异常综合征患者贫血、铁代谢、血脂及炎症指标等变化，以评价其治疗效果及安全性，为罗沙司他在低危骨髓增生异常综合征中的应用提供理论及实践数据。

## Abstract (en)

Myelodysplastic syndromes is a heterogeneous group of myeloid neoplastic disorders originating from hematopoietic stem cells and manifesting as pathological bone marrow hematopoiesis and a high risk of transformation to acute myeloid leukemia. In low-risk patients, the therapeutic goal is to improve hematopoiesis and quality of life. Roxadustat is the world's first oral small-molecule hypoxia-inducible factor prolyl hydroxylase inhibitor, which, unlike conventional erythropoietin, corrects anemia through various mechanisms. In this study, we retrospectively analyzed the changes in anemia, iron metabolism, lipids and inflammatory indexes in patients with low-risk myelodysplastic syndromes to evaluate its therapeutic efficacy and safety, and to provide theoretical and practical data for the application of roxadustat in myelodysplastic syndromes.

改善红细胞生成、消除贫血症状是大多数骨髓增生异常综合征（MDS）患者的主要治疗目标[1]。低危MDS（LR-MDS）患者的主要治疗目标是降低输血需求和避免反复输血治疗的负面影响[2]。尽管红细胞生成素（ESA）对很大一部分患者有效，总体耐受性良好，但其具有高度可变的红系反应率和高原发性耐药率[3]。罗特西普（Luspatercept）的出现，虽然改善了一部分LR-MDS患者的贫血症状，但临床使用较为不便[4]。罗沙司他是一种小分子低氧诱导因子（HIF）脯氨酰羟化酶（PHD）抑制剂，已在全球多个国家被批准用于治疗肾性贫血[5]。目前仅有一项国外的3期临床试验报道了罗沙司他用于治疗LR-MDS，且国内外仍缺乏系统性的疗效和安全性评估。本研究中，我们通过对比分析罗沙司他使用前后LR-MDS患者的贫血、铁代谢、血脂及炎症指标等变化，评价罗沙司他对LR-MDS患者的治疗效果及安全性，为罗沙司他在LR-MDS中的研究提供参考。

## 病例与方法

一、病例资料

回顾性分析自2020年7月至2022年11月就诊于南通大学附属医院血液内科的符合纳入标准的21例LR- MDS患者临床资料。入选标准：（1）入组患者或患者的亲属完全知情同意并签署知情同意书；（2）年满18周岁；（3）平均HGB水平（最近2次筛选评估）≤100 g/L；（4）修订版国际预后评分系统（IPSS-R）评分≤3分。排除标准：（1）合并严重心、肝、肾等脏器功能不全及其他免疫性相关性疾病；（2）MDS 5q−患者；（3）合并其他恶性肿瘤或消耗性疾病者；（4）合并先天性疾病或急性感染者；（5）对本研究所采用药物过敏者；（6）合并心理、精神疾病者；（7）临床资料不完整。本研究获南通大学附属医院伦理委员会的批准（批件号：2021-k075-02）。

二、治疗方案

罗沙司他每次100 mg口服，每周3次，连续治疗8周。治疗过程中允许使用口服补充铁剂治疗，但禁止静脉铁剂治疗。其中11例患者单用罗沙司他治疗，10例患者采用罗沙司他联合ESA治疗。

三、相关定义及标准

本研究采用MDS IWG 2006年提出的红系反应标准定义有效性[6]。MDS的分型标准及风险分层参照《骨髓增生异常综合征中国诊断与治疗指南（2019年版）》[7]。

四、观察指标

收集所有患者的一般资料，包括：年龄、性别、体重、血压、骨髓原始细胞比例、染色体核型、MDS诊断分型、确诊MDS病程等。记录患者治疗前及治疗后 8周RBC、HGB、红细胞比容、平均红细胞体积、平均血红蛋白含量、平均血红蛋白浓度、网织红细胞计数、网织红细胞百分比、促红细胞生成素（EPO）、C反应蛋白（CRP）、电解质、肾功能、肝功能、脂代谢及铁代谢指标。

五、统计学处理

采用SPSS 26.0软件对数据进行分析。符合正态分布的计量资料采用平均值±标准差表示，不符合正态分布的计量资料采用中位数（*IQR*）表示，治疗前后的组间比较分别应用配对*t*检验或配对Mann-Whitney *U*检验分析。计数资料采用率和百分比来表示，组间比较应用卡方检验或Fisher确切概率法分析。双侧*P*<0.05为差异有统计学意义。

## 结果

一、一般资料

本研究共纳入21例LR-MDS患者，其中女8例（38.10％），男13例（61.90％），中位年龄67（45～81）岁。骨髓原始细胞2.5％（0～4％），中位IPSS-R风险评分3（2.5～3.5）分。WHO分型：MDS-SLD 16例（76.19％），MDS-MLD 4例（19.05％），MDS-RS+ 1例（4.76％）。MDS的中位病程为14（12～22）个月。基线中位HGB水平为70（60～83）g/L。基线中位EPO水平为243（83～758）U/L，47.62％的患者EPO水平<200 U/L。

二、有效性

罗沙司他治疗8周后11例有效，有效率为52.38％。其中，15例HGB<80 g/L组患者7例（46.7％）有效，6例HGB 80～<100 g/L组患者4例（66.7％）有效，两组之间差异无统计学意义（Fisher，*P*＝0.635）。10例EPO<200 U/L组患者4例（40.0％）有效，11例EPO≥200 U/L组患者7例（63.6％）有效。6例CRP<5 mg/L组患者3例（66.7％）有效，15例CRP≥5 mg/L组患者7例（46.7％）有效。10例血清铁蛋白（SF）<1 000 µg/L组患者6例（60.0％）有效，11例SF≥ 1 000 µg/L组患者5例（45.5％）有效。根据既往是否使用ESA治疗进行亚组分析，两组差异均无统计学意义（均*P*>0.05）。

三、治疗前后贫血相关指标比较

在21例患者中，19例罗沙司他治疗2个月后HGB水平升高，治疗后较治疗前升高10（4～17）g/L，治疗后HGB水平最多能升高53 g/L；14例治疗后EPO水平降低，治疗后平均EPO水平较基线水平降低95 U/L（表1）。

**表1:** 21例低危骨髓增生异常综合征患者罗沙司他治疗前后贫血指标比较

指标	治疗前	治疗后	*P*值
Ret（×10^9^/L，*x*±*s*）	0.05±0.05	0.06±0.05	0.147
Ret%[%，*M*（范围）]	1.93（0.46~3.78）	2.54（0.69~3.41）	0.590
HGB[g/L，*M*（范围）]	70（60~83）	76（71~89）	0.001
RBC（×10^12^/L，*x*±*s*）	2.26±0.45	2.67±0.58	0.002
HCT（L/L，*x*±*s*）	0.22±0.04	0.26±0.06	<0.001
MCV[fl，*M*（范围）]	96.80（93.75~104.20）	98.80（93.10~102.25）	0.126
MCH[pg，*M*（范围）]	31.70（30.05~33.15）	31.60（30.60~33.40）	0.498
MCHC（g/L，*x*±*s*）	324±12	322±12	0.245
EPO[U/L，*M*（范围）]	243（83~758）	148（61~758）	0.109

注 Ret：网织红细胞计数；Ret％：网织红细胞百分比；HCT：红细胞压积；MCV：平均红细胞体积；MCH：平均血红蛋白含量；MCHC：平均血红蛋白浓度；EPO：促红细胞生成素

四、治疗前后其他相关指标比较

治疗后铁代谢指标变化不明显，11例患者转铁蛋白水平较前增高，12例患者总铁结合力较前增高（表2）。脂代谢指标方面，18例患者在使用罗沙司他治疗后总胆固醇（TCHO）水平较前降低，治疗后较治疗前降低1.00（1.20～0.25）mmol/L（表3）。治疗前后生化指标也未见明显变化（表4）。

**表2:** 21例低危骨髓增生异常综合征患者罗沙司他治疗前后炎症及铁代谢指标比较

指标	治疗前	治疗后	*P*值
SF[ng/ml，*M*（范围）]	1017（581~1580）	696（522~2000）	0.845
TRF（mg/L，*x±s*）	1646.2±458.3	1559.9±511.5	0.383
SI（µmol/L，*x±s*）	27.71±9.73	23.09±11.86	0.014
UIBC（µmol/L，*x±s*）	11.68±12.94	16.47±16.18	0.151
TIBC（µmol/L，*x±s*）	35.57±9.04	36.41±10.28	0.574
TSAT（%，*x±s*）	69.81±24.43	64.49±32.10	0.394
CRP[mg/L，*M*（范围）]	8（3~11）	5（3~9）	0.305

注 SF：血清铁蛋白；TRF：转铁蛋白；SI：血清铁；UIBC：不饱和铁结合力；TIBC：总铁结合力；TSAT：转铁蛋白饱和度；CRP：C反应蛋白

**表3:** 21例低危骨髓增生异常综合征患者罗沙司他治疗前后脂代谢指标比较

指标	治疗前	治疗后	*P*值
TCHO[mmol/L，*M*（范围）]	2.90（2.45~3.90）	2.20（1.75~3.00）	0.009
TG[mmol/L，*M*（范围）]	1.04（0.77~1.33）	0.98（0.63~1.66）	0.702
HDL-c[mmol/L，*M*（范围）]	0.80（0.73~0.92）	0.79（0.66~0.93）	0.862
LDL-c（mmol/L，*x±s*）	1.96±0.78	1.90±0.73	0.629

注 TCHO：总胆固醇；TG：甘油三脂；HDL-c：高密度脂蛋白胆固醇；LDL-c：低密度脂蛋白胆固醇

**表4:** 21例低危骨髓增生异常综合征患者罗沙司他治疗前后生化指标比较

指标	治疗前	治疗后	*P*值
K^+^[mmol/L，*x±s*）	4.02±0.99	4.05±0.97	0.838
Na^+^[mmol/L，*x±s*）	138±3	139±4	0.318
Cl^−^[mmol/L，*M*（范围）]	106（103~108）	106（103~108）	0.706
Ca^2+^[mmol/L，*M*（范围）]	2.10（1.98~2.19）	2.11（2.02~2.19）	0.365
ALT[U/L，*M*（范围）]	19（15~31）	20（15~28）	0.150
AST[U/L，*M*（范围）]	17（10~23）	16（10~24）	0.931
LDH[U/L，*M*（范围）]	208（162~766）	260（192~482）	0.313
Scr[µmol/L，*M*（范围）]	68（61~91）	72（53~88）	0.454
UA[µmol/L，*M*（范围）]	366（294~463）	409（304~489）	0.455

注 LDH：乳酸脱氢酶；Scr：肌酐；UA：尿酸

五、安全性分析

在8周治疗期间里，无一例患者因不良反应退出试验，也无一例死亡或进展。5例（23.81％）患者出现恶心，4例（19.05％）腹泻，2例（9.52％）头晕，没有观察到血栓及心血管事件的发生。

## 讨论

本项回顾性研究主要疗效分析显示，罗沙司他的有效率为52.38％。根据Meta分析结果，ESA治疗LR-MDS的红系反应率为38％～72％[8]。ESA的反应率受EPO水平影响，EPO低水平的患者往往表现出更高的应答率[9]。3期临床试验结果显示，EPO<200 U/L和EPO≥200 U/L的患者反应率分别为45％和5％[10]。EPO高水平的患者对ESA反应不佳。在另一项3期临床试验中，EPO>200 U/L的患者没有表现出血液学改善[11]。

除了EPO外，铁是红细胞生成最重要的参与者，是合成HGB的关键原料。在正常人体内，铁的吸收和代谢可以通过调节作用使体内的铁含量保持平衡，而在依赖输血治疗的LR-MDS患者体内，这种动态平衡被打破[12]–[13]。反复输注红细胞会导致体内铁含量剧增，增加铁负荷，造成铁过载[14]。在本研究中，治疗前后铁代谢指标无明显变化。MDS患者由于骨髓病态造血、无效造血、免疫功能低下，常并发炎症感染。炎症过程可能也是MDS的致病驱动因素[15]。在本研究中，不同基线CRP水平的患者在治疗8周后，达标率差异无统计学意义（*P*>0.05）。

罗沙司他除了纠正贫血外，在脂代谢方面的作用也值得关注。在我们的研究中，治疗后TCHO水平较基线水平下降（*P*<0.05），这与先前的研究结果相一致[11]。罗沙司他的降脂作用机制目前尚不明确，可能部分与HIF介导乙酰辅酶A和3-羟基-3-甲基戊二酰辅酶A还原酶（胆固醇合成中的限速酶）的降解有关[16]–[17]。由于高血脂是心血管事件的危险因素之一，罗沙司他的降血脂作用可能有利于减少心血管事件的发生，但仍需进一步的探索。

在不良反应方面，我们观察到罗沙司他治疗前后患者的电解质、肝功能、肾功能指标差异无统计学意义（均*P*>0.05）。通过随访，我们观察到，有5例（23.81％）患者出现恶心，4例（19.05％）患者出现腹泻，这与先前报道的罗沙司他易导致胃肠道不良反应的结果一致[18]。在本研究中没有观察到血栓及心血管事件的发生，这可能需要更长的随访。

综上所述，罗沙司他可用于改善LR-MDS患者的贫血，可以作为EPO高水平患者的治疗选择。本研究存在一定的局限性，在未来的研究中，我们将进行多中心随机重复性试验，并进行更长时间的随访观察。
